# An injectable gelatin/sericin hydrogel loaded with human umbilical cord mesenchymal stem cells for the treatment of uterine injury

**DOI:** 10.1002/btm2.10328

**Published:** 2022-05-18

**Authors:** Lixuan Chen, Ling Li, Qinglin Mo, Xiaomin Zhang, Chaolin Chen, Yingnan Wu, Xiaoli Zeng, Kaixian Deng, Nanbo Liu, Ping Zhu, Mingxing Liu, Yang Xiao

**Affiliations:** ^1^ Guangzhou University of Chinese Medicine Guangzhou Guangdong China; ^2^ Jinshazhou Hospital of Guangzhou University of Chinese Medicine Guangzhou Guangdong China; ^3^ Jiangmen Maternity and Child Health Care Hospital Jiangmen Guangdong China; ^4^ Translational Medicine Center The Second Affiliated Hospital of Guangzhou Medical University Guangzhou Guangdong China; ^5^ National Seed Cell Bank of South China for Tissue Engineering Guangzhou Guangdong China; ^6^ Department of Gynecology, Shunde Hospital Southern Medical University (The First People's Hospital of Shunde) Foshan Guangdong China; ^7^ Guangdong Cardiovascular Institute, Guangdong Provincial People's Hospital Guangdong Academy of Medical Sciences Guangzhou China; ^8^ Department of Obstetrics and Gynecology, Key Laboratory for Major Obstetric Diseases of Guangdong Province The Third Affiliated Hospital of Guangzhou Medical University Guangzhou Guangdong China; ^9^ Shenzhen Qianhai Shekou Pilot Free Trade Zone Hospital, Shekou Shenzhen Guangdong China

**Keywords:** fertility restoration, methacrylate gelatin (GelMA), methacrylate sericin (SerMA), regeneration, stem cells, uterine injury

## Abstract

Abnormal endometrial receptivity is a major cause of the failure of embryo transplantation, which may lead to infertility, adverse pregnancy, and neonatal outcomes. While hormonal treatment has dramatically improved the fertility outcomes in women with endometriosis, a substantial unmet need persists in the treatment. In this study, methacrylate gelatin (GelMA) and methacrylate sericin (SerMA) hydrogel with human umbilical cord mesenchymal stem cells (HUMSC) encapsulation was designed for facilitating endometrial regeneration and fertility restoration through in situ injection. The presented GelMA/10%SerMA hydrogel showed appropriate swelling ratio, good mechanical properties, and degradation stability. In vitro cell experiments showed that the prepared hydrogels had excellent biocompatibility and cell encapsulation ability of HUMSC. Further in vivo experiments demonstrated that GelMA/SerMA@HUMSC hydrogel could increase the thickness of endometrium and improve the endometrial interstitial fibrosis. Moreover, regenerated endometrial tissue was more receptive to transfer embryos. Summary, we believed that GelMA/SerMA@HUMSC hydrogel will hold tremendous promise to repair or regenerate damaged endometrium.

## 1 INTRODUCTION

The uterus is one of the most important reproductive organs for women, and the endometrium (as the place for implantation, embryonic development, and pregnancy maintenance of female fertilized eggs) is the key factor for the success of pregnancy.^1^, ^2^ However, endometrial damage was caused by physical injury (induced abortion, frequent uterine surgery) and biochemical injury (infection, endocrine disrupting chemicals), which could lead to impaired endometrial proliferation and affect embryo implantation and implantation, resulting in female infertility or repeated abortion.^3^, ^4^, ^5^ Currently, there are still big challenges for promoting the repair and regeneration of endometrium after injury. For the regeneration and repair of endometrial injury, the traditional treatment mainly includes surgical treatment, intrauterine barrier, endocrine therapy, increasing endometrial blood perfusion, and so on.^6^, ^7^ However, the methods to promote endometrial regeneration after the moderate and severe injury are still very limited, which could not avoid endometrial regeneration disturbance and postoperative adhesion recurrence. Therefore, the key to successful treatment is the ability to prevent the reformation of uterine adhesion and promote the regeneration and repair of endometrium.

Stem cells have the pluripotency, the ability to release growth factors and regulate inflammation, which showed great potential in the treatment of a variety of injuries and diseases in regenerative medicine.^8^, ^9^, ^10^ Mesenchymal stem cells (MSCs, a kind of adult stem cells), which have the ability of stem cell proliferation and multi‐directional differentiation, which could be isolated from a variety of tissues (such as umbilical cord, endometrial polyps, menstrual blood, bone marrow, adipose tissue, etc).^11^, ^12^, ^13^ Currently, a large number of animal experiments showed that MSC could improve the repair of endometrial injury and increase the pregnancy rate.^14^, ^15^ In the mouse model of endometrial injury, intraperitoneal transplantation of endometrial mesenchymal stem cells (EnMSC) could effectively repair the damaged endometrium, increase the uterine microangiogenesis, and improve the pregnancy rate.^16^ Prior study proposed that intravenous injection of human umbilical cord mesenchymal stem cells (HUMSC) could increase the thickness and glands of endometrium, increase the implantation rate of embryos, reduce the excessive fibrosis, promote the vascular growth and endothelial cell proliferation, regulate the inflammatory factors, and restore the structure and function of ethanol‐damaged endometrium.^3^ Moreover, HUMSC have the characteristics of low immunogenicity, high ability of self‐replication, noninvasive collection, which could promote the regeneration and repair of injured tissue. However, there are still some shortcomings in the clinical application of MSC therapy, such as the tumorigenic potential of stem cells, thrombosis, fever, and other adverse reactions.^17^, ^18^ Meanwhile, the low efficiency of cell implantation caused by local injection greatly limited the clinical promotion of stem cell therapy.^19^ One of the main reason for the low cell retention rate is the lack of three‐dimensional matrix to support the survival, migration, and development of transplanted cells.^20^

To resolve these problems, a variety of injectable hydrogel systems have been developed, which could provide mechanical protection to prevent cell membrane from being destroyed during injection and form a stable network after injection, resulting in promoting cell adhesion and growth.^21^, ^22^, ^23^, ^24^, ^25^ However, many available injectable hydrogels have some problems such as poor mechanical properties, low cell survival rate, or inability to accurately control their gelation process and gel properties.^26^, ^27^ Prior study reported that the natural extracellular matrix (ECM) environment played a key role in influencing through a series of complex physical, mechanical, and biochemical signals.^28^, ^29^ To further understand the regulation of cell behavior by ECM, designing new materials with precise adjustable structure, mechanical properties, biodegradability, and cell interaction has been a challenging task for the research community. Therefore, our goal is to design an injectable and adjustable hydrogel crosslinked by blue or ultraviolet light to meet the different requirements of cell culture and tissue engineering applications.

Gelatin (Gel) is a derivative of collagen, which is often applied in hydrogels because of its good gelling, biocompatibility, and biodegradability.^30^ However, one limitation of gelatin‐based hydrogels is poor mechanical properties and thermal stability.^31^ These limitations can be overcome by chemical or physical cross‐linking of gelatin. In addition, the addition of other ECM components can enrich gelatin hydrogels. Sericin (Ser) is a main component of natural silk and has good water solubility and excellent biocompatibility.^32^, ^33^ Meanwhile, sericin can promote cell adhesion and proliferation, antioxidant, and inhibit tyrosinase activity.^34^ These advantages make sericin gradually become the research focus of new natural materials in the field of tissue engineering and regenerative medicine.^35^ In addition, the interpenetrating polymer network (IPN) hydrogel is a unique structure, which allows two independent networks to combine with each other (maintain the required properties of the original polymer) to obtain better mechanical properties.^36^, ^37^ In this study, we hypothesized that the modified gelatin and sericin could effectively cross‐link into IPN hydrogel structure, which is suitable for HUMSC cells to be incorporated into the injured tissue and play a role in tissue repair.

**SCHEME 1:**
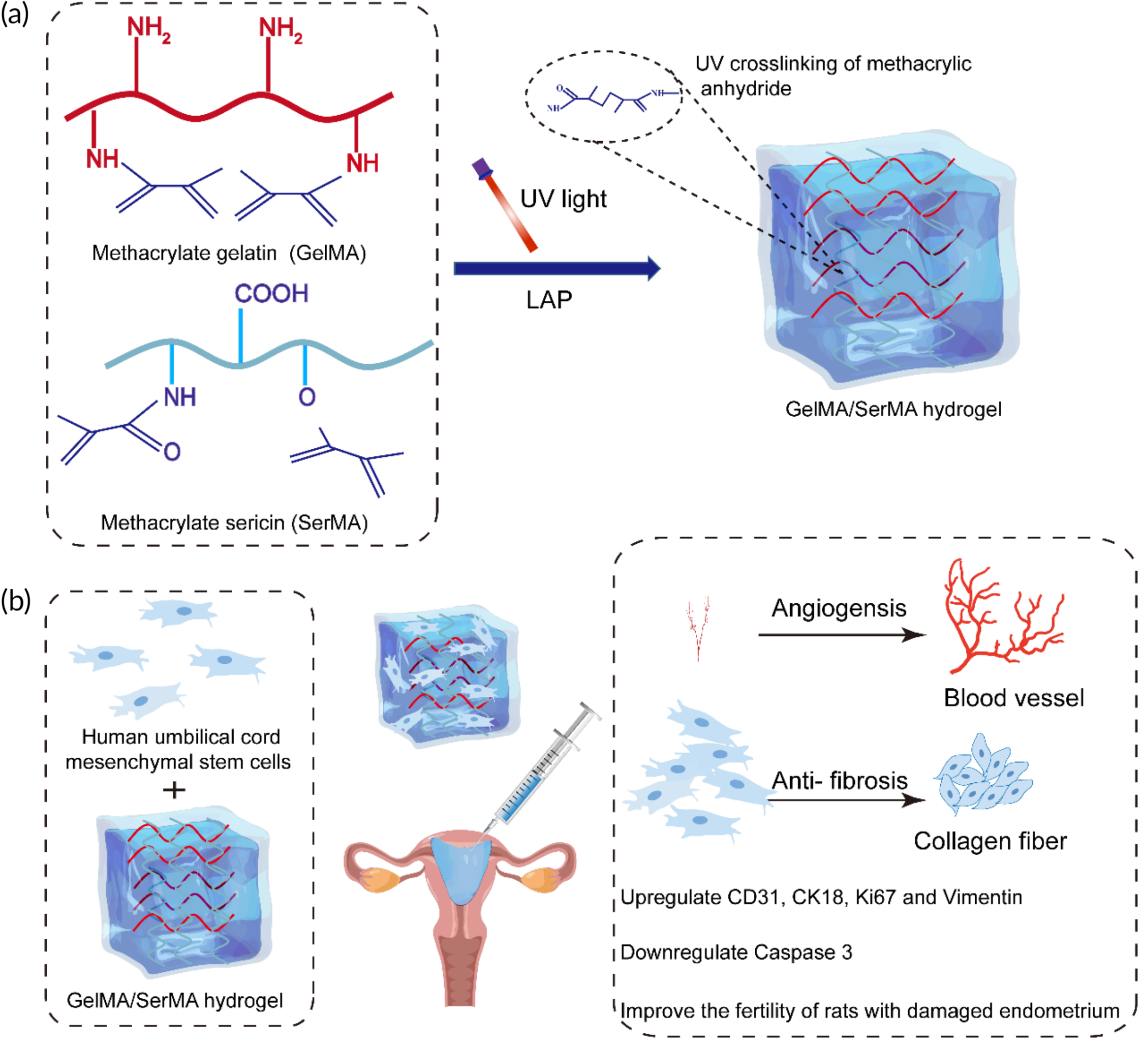
(a) Preparation of GelMA/SerMA hydrogel. (b) GelMA/SerMA hydrogel system encapsulating HUMSC for the treatment of uterine injury by injection into the uterine cavity

In this study, we aim to develop an injectable hydrogel based on methacrylate gelatin (GelMA) and methacrylate sericin (SerMA) matrix as cell delivery carriers of HUMSC to promote endometrial repair (Scheme 1). The porous structure, swelling properties, mechanical properties, and degradation properties of this hydrogel were characterized. In addition, the cytocompatibility of hydrogels was studied in detail by CCK‐8 and living/dead cell staining using the L929 and HUMSC cells. Meanwhile, cell encapsulation ability was characterized by three‐dimensional live dead staining and cytoskeleton staining. And excellent therapeutic effect for promoting the endometrial repair and fertility restoration were shown in vivo experiments. In summary, all of these results demonstrated that the GelMA/SerMA@HUMSC hydrogel will have great potential in the repair of endometrial injury.

## 2 EXPERIMENTAL SECTION

### 2.1 Materials

The silkworm cocoon was obtained from the Sericultural Research Institute of Guangdong Academy of Agricultural Sciences (Guangzhou, China). Gelatin (adhesive strength ~500 g bloom) was purchased from the Sigma‐Aldrich Chemical Company (Shanghai, China). Lithium bromide and methacrylic anhydride (MA) were obtained from Macklin Biochemical Technology Co., Ltd. (Shanghai, China). Lithium phenyl‐2,4,6‐trimethylbenzoylphosphinate (LAP) was obtained from Suzhou Intelligent Manufacturing Research Institute (Suzhou, China). Human umbilical cord mesenchymal stem cells (HUMSC, AC340316) was purchased from American Type Culture Collection (Manassas, VA). Cell Counting Kit‐8 (CCK‐8), Live/Dead cell staining kits, Alexa Flour 488 phalloidin and 2‐(4‐Amidinophenyl)‐6‐indolecarbamidine dihydrochloride (DAPI) were obtained fromn Biyuntian Biotechnology CO., Ltd. (Shanghai, China). All the chemicals were analytically pure, which was used without further purification.

### 2.2 Synthesis of methacrylated gelatin (GelMA)

The preparation of GelMA follows the reported method in the literature with slight modification.^38^ Briefly, 10 g of gelatin was dissolved 100 ml of 50°C preheated deionized water. Then, 6 ml of MA reagent was slowly dripped into the gelatin solution, the final solution was stirred for 12 h. The reaction was performed in darkness, a white milky solution was obtained after the completion of the reaction. The obtained solution was centrifuged for at 6000 rpm for 5 min and the supernatant was dialyzed in a dialysis bag (14‐kDa molecular weight cutoff) against distilled water at 40°C for 6 days. Finally, the solution was freeze‐dried to obtain white porous foam‐like GelMA prepolymer and stored it in a refrigerator at −20°C for later use.

### 2.3 Synthesis of methacrylated sericin (SerMA)

Sericin was extracted from natural silkworm cocoon through the high heat/alkaline degumming method according to a previously reported procedure with minor modifications.^39^ Briefly, 40 g of cocoons were cut into small pieces and boiled in Na_2_CO_3_ solution (1 L, 0.02 M) for 1 h. Then, the insoluble residue was removed by miracloth and the resultant solution was dialyzed (MWCO, 3500 Da) against distilled water for 3 days. Sericin was then obtained by lyophilization.

SerMA was synthesized following previously published protocols.^40^ A 10 g of sericin was dissolved 100 ml of deionized water. Then, 6 ml of MA reagent was slowly dripped into the sericin solution and the pH was adjusted to 9.0 with 2 M NaOH during the reaction. After stirring for 1 h at room temperature, the pH of the solution was stabilized at 7.0 using 1 M HCl. Then, the insoluble residue was removed by miracloth and the resultant solution was dialyzed (MWCO, 3500 Da) against distilled water for 3 days. Finally, SerMA are obtained by lyophilization and stored it in a refrigerator at −20°C for later use.

### 2.4 Preparation of GelMA/SerMA hydrogel

The concentration of GelMA was fixed at 20% (w/v) in deionized water, and varying amounts of SerMA (5%, 10%, and 15% [w/v]) were dissolved in deionized water. The two solutions were then mixed at a volume ratio 1:1, and LAP was added at 0.1% (w/v). The mixtures were exposed to a UV light (365 nm) at a power density of 5 mW/cm^2^ for 30 s to produce GelMA/SerMA hydrogels.

### 2.5 Characterization of polymers

The chemical structure of GelMA and SerMA was characterized by ^1^H NMR (Bruker 400 MHz NMR spectrometer). The chemical construction of GelMA and SerMA was characterized by Fourier transform infrared spectroscopy (FTIR; ThermoScientific, Waltham, USA). The micromorphology of GelMA and GelMA/SerMA hydrogels was observed by scanning electron microscopy (SEM; S‐3400, Hitachi, Japan) with an accelerating voltage of 5 kV. The averaged pore diameter of was calculated using Nano measure software.

### 2.6 Physical properties of hydrogels

#### 2.6.1 Swelling ratio

The swelling ratio of the composite hydrogel was investigated using a quality method.^41^ Briefly, 400 μl of hydrogel was immersed in phosphate buffered solution (PBS) (2 ml, pH 7.4) and incubated at 37°C in an incubator. At indicated time points (2, 4, 6, 8, and 24 h), the hydrogel was taken out and weighted after wiping off all surface water with a filter paper. The swelling ratio (%) was calculated based on the following formula:

$$\text{Swelling ratio}=\frac{\left(W_{t}-W_{0}\right)}{W_{0}}\times 100$$

where W_0_ represents the initial weight of the hydrogel and W_t_ means the weight of the swollen hydrogel at time t.

#### 2.6.2 Rheological tests

Rheological measurements of the composite hydrogel were performed using a rotated rheometer (AR 2000ex; TA Instrument, USA). In the time testing, the changes of the storage modulus (*G*') and loss modulus (*G*") were registered over time with the frequency of 1 Hz and the shear strain of 1%. In the frequency testing, the range of frequency was 0.1–10 Hz with a shear strain of 1%.

#### 2.6.3 Compression test

Compression experiments were conducted on a universal testing machine (Instron5543A; Boston, USA). A volume of 600 μl of hydrogel solutions were cured for 30 s in a 48‐well plate and placed at the center of the lower compression plate. The compression rate of 1 mm/min was applied, and strain level was up to ≈40% of the original height.

#### 2.6.4 In vitro biodegradation

The degradation of hydrogels in vitro was measured following the method reported in the literature with slight modification.^42^, ^43^ Briefly, the prepared hydrogels were incubated PBS solution (0.01 mol/L, pH = 7.4) with or without 1000 U/ml of lysozyme at 37°C with stirring speed of 60 rpm. At indicated time points, the degraded hydrogels were taken out, washed with deionized water, weighted after freeze‐drying. The weight loss rate was calculated using the following formula:

$$\text{Weight loss rate}\;\left(\%\right)=\frac{W_{t}}{W_{0}}\times 100$$

where W_0_ represents the original weight of the lyophilized hydrogel and W_t_ means the weight of the lyophilized hydrogel after degradation time.

### 2.7 In vitro biocompatibility assessment

L929 and HUMSC cells were used to evaluate the biocompatibility of the hydrogel materials. L929 cells were cultured in high‐glucose Dulbecco's modified eagle medium (DMEM) supplemented with 10% FBS and 1% (v/v) penicillin/streptomycin at 37°C in 5% CO_2_. HUMSC cells were cultured in DMEM/F12 medium supplemented with 10% FBS and 1% (v/v) penicillin/streptomycin at 37°C in 5% CO_2_.

Hydrogel samples were passed through a 0.22 μm cell filter. Hydrogels of 200 μl were placed on the 48‐well plate and then exposed to 405  nm blue light for 10 s. Next, 500 μl cell suspensions of L929 (2 × 10^4^ cells/ml) and HUMSC (2 × 10^4^ cells/ml) were added onto the hydrogel surface cultured in the corresponding medium, respectively. After 1, 3, and 5 days culture, the cells were incubated with CCK‐8 solution for 2 h to evaluate the cell viability of hydrogel groups. Live/Dead cell staining kit was performed according to manufacturer's instructions to evaluate the survival and growth of L929 and HUMSC cells in hydrogels, respectively. Meanwhile, SEM experiments of HUMSC cultured on GelMA/SerMA hydrogels at 1, 3, and 5 days were performed according to a previously reported procedure with minor modifications.^44^

### 2.8 Scratch assays

HUMSC cells were seeded in 24‐well plates at a density of 2 × 10^4^ cells/well to create a confluent monolayer. Then, cells were starved in serum‐free medium for 24 h, and a 200‐μl pipette tip was used to scratch the monolayer. Next, the cells were washed with PBS to remove debris and supplied with 500 μl of leach liquor (hydrogel: medium = 1:10, mass ratio). At each indicated time interval, cell migration was monitored and photographed. For quantification, the surface area of the scratch was measured at each time‐point using IPP 6.0 software.

### 2.9 HUMSC cells encapsulation

#### 2.9.1 Live/Dead cell imaging and viability

Briefly, 100 μl of cell suspension of HUMSC (5 × 10^6^ cells/ml) in the logarithmic growth phase were added to 900 μl of GelMA and GelMA/SerMA pregel solution, respectively. The samples were mixed well by pipetting, placed on the 12‐well plate, and then exposed to 405 nm blue light for 10 s. Next, the cell‐encapsulated hydrogels were incubated in DMEM/F12 medium. After 1, 4, and 7 days culture, Live/Dead cell staining kit was used to evaluate the survival and growth of the encapsulated cells. Fluorescence images were captured using a confocal microscope (FV 3000; Olympus, Japan), and 3D reconstructed images were performed with Imaris 7.2.1 software. CCK‐8 assay was used to evaluate the viability of the encapsulated cells.

#### 2.9.2 Cytoskeleton staining

The cell‐encapsulated hydrogels were washed three times with PBS and then fixed in 4% paraformaldehyde for 0.5 h. Then, the samples were washed three times in PBS for 5 min, permeabilized in 0.5% Triton‐X‐100 for 10 min, and rinsed three times in PBS for 5 min. Next, the cell‐encapsulated hydrogels were incubated Tetramethyl rhodamine isothio‐cyanate phalloidin for 1 h and DAPI for 10 min while protected from light. Fluorescence images were captured using a confocal microscope (FV 3000; Olympus).

### 2.10 Animal model

#### 2.10.1 Establishment of mice endometrial damage model in vivo

All animal protocols were reviewed and approved by the Institutional Animal Care and Use Committee (IACUC) of Jinan University. Briefly, all female mice were anesthetized by intraperitoneal injection of 60  mg/kg pentobarbital. The vaginal opening of mice was perfused with PBS (Sham operation group) and 95% ethanol for 30 s (experimental groups). Next, the uterus of experimental groups was injected with 100 μl PBS (normal group), GelMA/SerMA hydrogel and HUMSC cells encapsulated GelMA/SerMA hydrogel (1 × 10^6^ cells), respectively. Then the uterus was exposed to 405 nm blue light for 30  s. The mice were euthanized and uterine tissues were excised and sectioned or frozen at Day 21 in the diestrous stage of three estrus cycles.

#### 2.10.2 Estrous cycles observation

The estrous cycles of the mice were monitored daily. The vagina of the mice was aspirated and washed three times with 20 μl saline through a 200 μl pipette tip. Then, the above saline was directly smeared onto glass microscope slides to air dry. Giemsa A solution was added to stain the fixed cells for 30 s, and Giemsa B solution was added to stain the fixed cells for 2 min. Glass slides were washed well in distilled water, dried on a warmer at 50°C, imaged by microscopy (RM2016; Leica, Shanghai, China).

#### 2.10.3 Histological examination

The uterine tissues were fixed in 4% paraformaldehyde solution overnight, followed by dimethylbenzene, and embedded in paraffin. All tissue blocks were cut into serial 4 μm thick sections and stained with stained with hematoxylin and eosin (H&E) and Masson's trichrome staining. Immunohistochemistry of TGF‐β1 was performed according to the previous steps.^45^ The pictures of the stained sections were captured using a microscope (RM2016; Leica).

#### 2.10.4 Immunofluorescence staining

After paraffin sections rehydrated, 4 μm‐thick sections were blocked using 5% BSA, after which they were incubated with mouse anti‐CD31 (Servicebio, GB13428, 1:1000), mouse anti‐CK18 (Servicebio, GB11232, 1:1000), mouse anti‐Ki67 (Servicebio, GB111141, 1:1000), mouse anti‐Vimentin (1:100; Abcam), and mouse anti‐Caspase 3 (Servicebio, GB11009‐1, 1:1000) at 4°C overnight. Next, sections were washed three times using PBS and incubated with HRP‐labeled goat anti‐rabbit IgG secondary antibody for 1 h at room temperature. Subsequently, the sections were washed with PBS and reacted with DAB solution. Nuclei were counterstained with DAPI. Images of sections were also collected by the inverted fluorescence microscope (TE2000‐S; Nikon, Japan). Images were analyzed using IPP 6.0 software to measure the percentage of positive (red color) pixels (positive staining area).

#### 2.10.5 Western blot analysis

For protein extraction, the samples were homogenized in tissue protein extraction reagent. Protein concentration of supernatants was determined by the BCA protein assay kit. The 20 mg of protein per sample was separated via 10% SDS‐PAGE and transferred onto PVDF membranes. Afterward, the proteins were then incubated overnight at 4°C with different primary anti‐bodies: anti‐CD31 (Bioss, bs‐0915R, 1:1000), anti‐CK18 (Servicebio, GB11232, 1:1000), anti‐Vimentin (Servicebio, GB1192, 1:1000) or anti‐Caspase 3 (Servicebio, GB11767C, 1:1000). Membranes were then washed three times with TBST and incubated with the HRP‐conjugated anti secondary antibody. The ImageJ software was used for densitometric analyses of protein bands.

### 2.11 Fertility evaluation

The fertility of each group of mice was evaluated after three estrous cycles. Briefly, the female mice naturally mated with male mice in cages, and the ratio of female‐to‐male mice was 2:1. At 16 days after the occurrence of vaginal suppository in female mice, the female mice were sacrificed to confirm whether they were pregnant or not.

### 2.12 Statistical analysis

All data were expressed as the average value ± standard deviation at least three independent analyses. The values of *p* values were calculated by independent‐sample t‐test or one‐way analyses of variance (ANOVA) test through statistical software SPSS19.0. A value of *p* < 0.05 was defined as statistically significant: * (*p* <  0.05), ** (*p* < 0.01), and *** (*p* < 0.001).

## 3 RESULTS AND DISCUSSION

### 3.1 Characterization of the hydrogel

**FIGURE 1:**
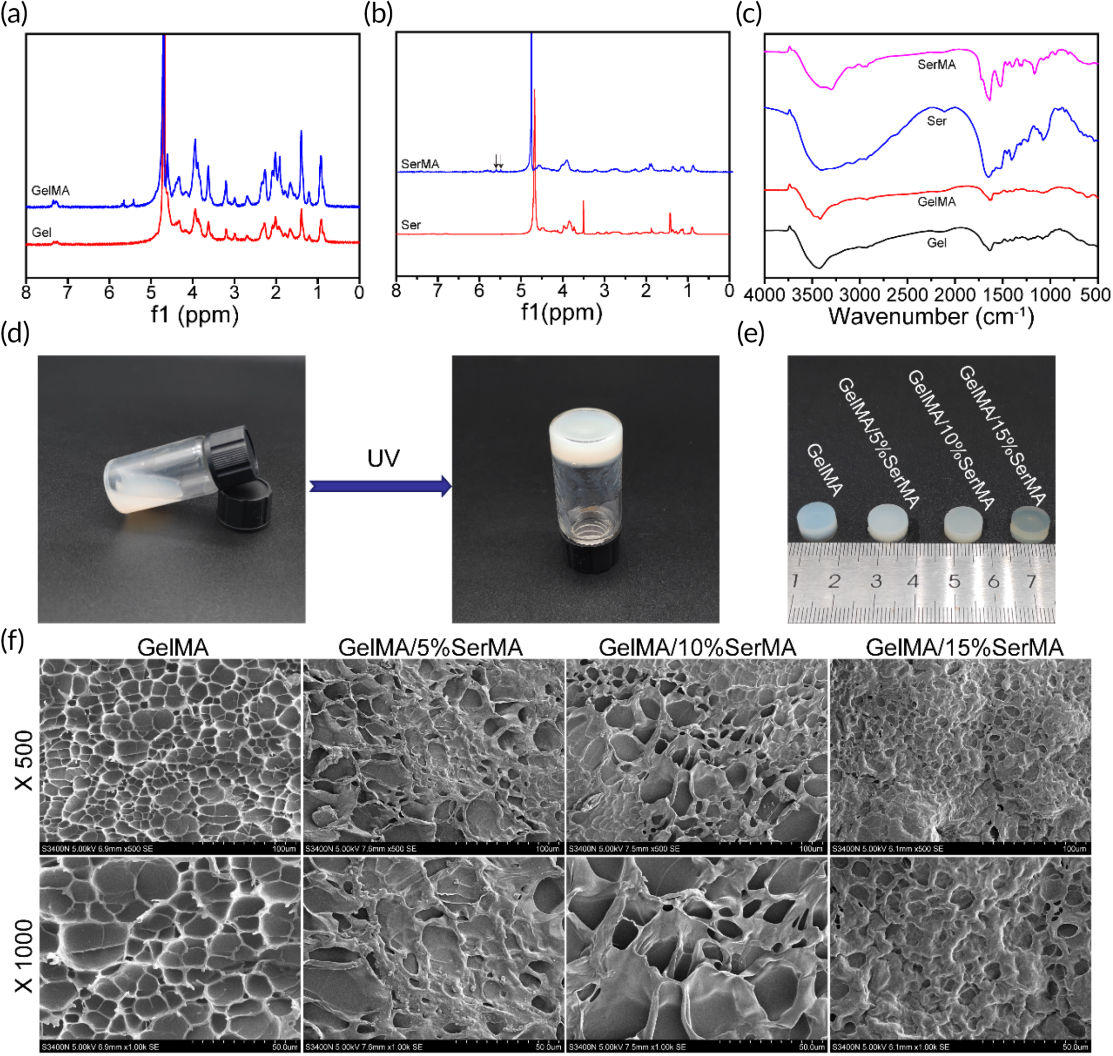
(a) ^1^H NMR spectra of Gel and methacrylate gelatin (GelMA). (b) ^1^H NMR spectra of Ser and SerMA. (c) Fourier‐transform infrared (FTIR) spectra of Gel, GelMA, Ser, and SerMA. (d) The synthesis procedures of GelMA/SerMA. (e) The appearances of cylindrical hydrogels. (f) Structural morphology of hydrogels

In order to form hydrogels for endometrial repair, methacrylate gelatin (GelMA) and methacrylate sericin (SerMA) polymers were synthesized through the reaction of methacrylic anhydride with gelatin and sericin. The ^1^H NMR spectrum of GelMA had two peaks at 5.42 and 5.65 ppm (Figure 1a), indicating that the methacrylic group was successfully grafted onto the molecular backbone of gelatin. The ^1^H NMR spectrum of SerMA had two peaks at 5.4 and 5.51 ppm (Figure 1b), verifying the methacrylic group was successfully grafted onto the molecular backbone of sericin.

As shown in Figure 1c, Fourier‐transform infrared spectroscopy (FTIR) spectra analysis was carried out for characterization of synthesized GelMA and SerMA. The spectrum of GelMA displayed the typical hydroxyl group peak at around 3249 cm^−1^. In addition, the characteristic peak at 1631, 1539, and 1444 cm^−1^ belonging to the C=O bond (amide I), bending of N—H bond (amide II), and plane vibration of C—N and N—H (amide III), respectively. Notably, the characteristic peaks of the amide II and amide III could be observed in FTIR spectrum of GelMA, which suggested the successful formation of GelMA. The spectrum of SerMA displayed that the amide I absorption peak at 1644 cm^−1^ shifted to 1637 cm^−1^.

As shown in Figure 1d, the synthesized GelMA/SerMA mixture hydrogel was flowing liquid before crosslinking, and gradually changes into solid phase after the UV curing. Figure 1e displayed the photographs of GelMA/SerMA with different concentration of SerMA, which were opalescent and transparent state with a smooth surface. The hydrogel morphology displayed a uniform porous network structure, which played an important role in the effective diffusion of nutrients and gases and provided a suitable moisture environment for cell growth (Figure 1f). The calculated pore size for hydrogels was 10.5 ± 6.3, 7.1 ± 5.3, 10.0 ± 9.9, and 5.1 ± 4.1 μm, respectively (Figure S1).

### 3.2 Swelling ratio assay

**FIGURE 2:**
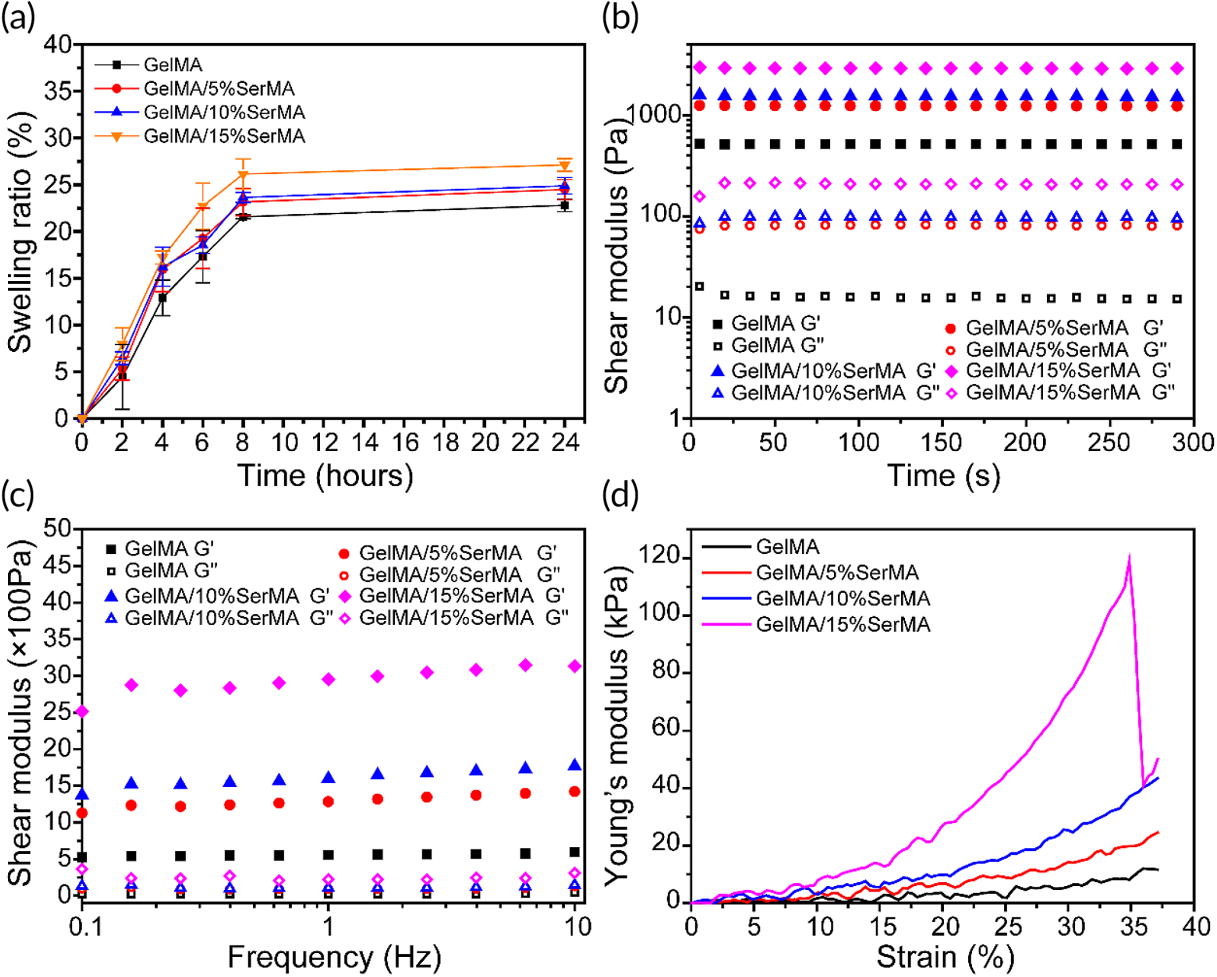
(a) The swelling ratio of hydrogels. (b) The rheological properties. (c) The hydrogel viscosity with frequency in a range between 0.1 and 100 Hz. (d) Compressive stress–strain curve

As shown in Figure 2a, the swelling degree of GelMA and GelMA/SerMA hydrogels increases with time and is stable in PBS from 8 to 24 h. All hydrogels became almost saturated after 8 h, and reach their equilibrium swelling. The swelling ratio of SerMA‐free GelMA, GelMA/10%SerMA, GelMA/15%HAMA, and GelMA/20%HAMA hydrogels were 22.8% ± 0.6%, 24.5% ± 1.1%, 24.8% ± 0.9%, and 27.1% ± 0.7%, respectively. These data showed that the prepared hydrogel will not expand due to absorbing too much water in the process of tissue application in vivo, thus avoiding the damage to the surrounding tissue. The addition of SerMA slightly increased the swelling rate of the hydrogel, which may be due to more holes in GelMA/SerMA hydrogel.

### 3.3 Rheological and compression analysis

Figure 2b shows the variation curve of the storage modulus (*G*') and the loss modulus (*G*") of hydrogels with time. It can be seen that both *G*' and *G*" do not change significantly with time, and *G*' has always been greater than *G*", indicating that the hydrogel has good stability. Meanwhile, the *G*' of the GelMA/SerMA hydrogel is higher than that of GelMA hydrogels, indicating that the stiffness of the GelMA/SerMA hydrogel is larger than that of GelMA hydrogels. Figure 2c showed the relationship between the *G*' and the *G*" of the hydrogel at the rotational speed of the rheometer tray at 0.1–10 Hz. It can be found that the *G*' of the four kinds of hydrogel materials is larger than *G*", which indicates that the hydrogel can stably maintain its solid state at this frequency.

An ideal hydrogel for endometrial repair should equip with good mechanical properties to keep its integrity during use.^46^ Figure 2d showed the test results of Young's compression modulus of hydrogel material under 40% strain. It can be found that GelMA/20%SerMA hydrogel has the largest compression modulus (118 kPa), which showed strong rigidity. Notably, the compression modulus of GelMA/10%SerMA was 40 kPa, which was more suitable for application of uterine injury and close to that of human tissues and organs.^47^

### 3.4 In vitro degradation

**FIGURE 3:**
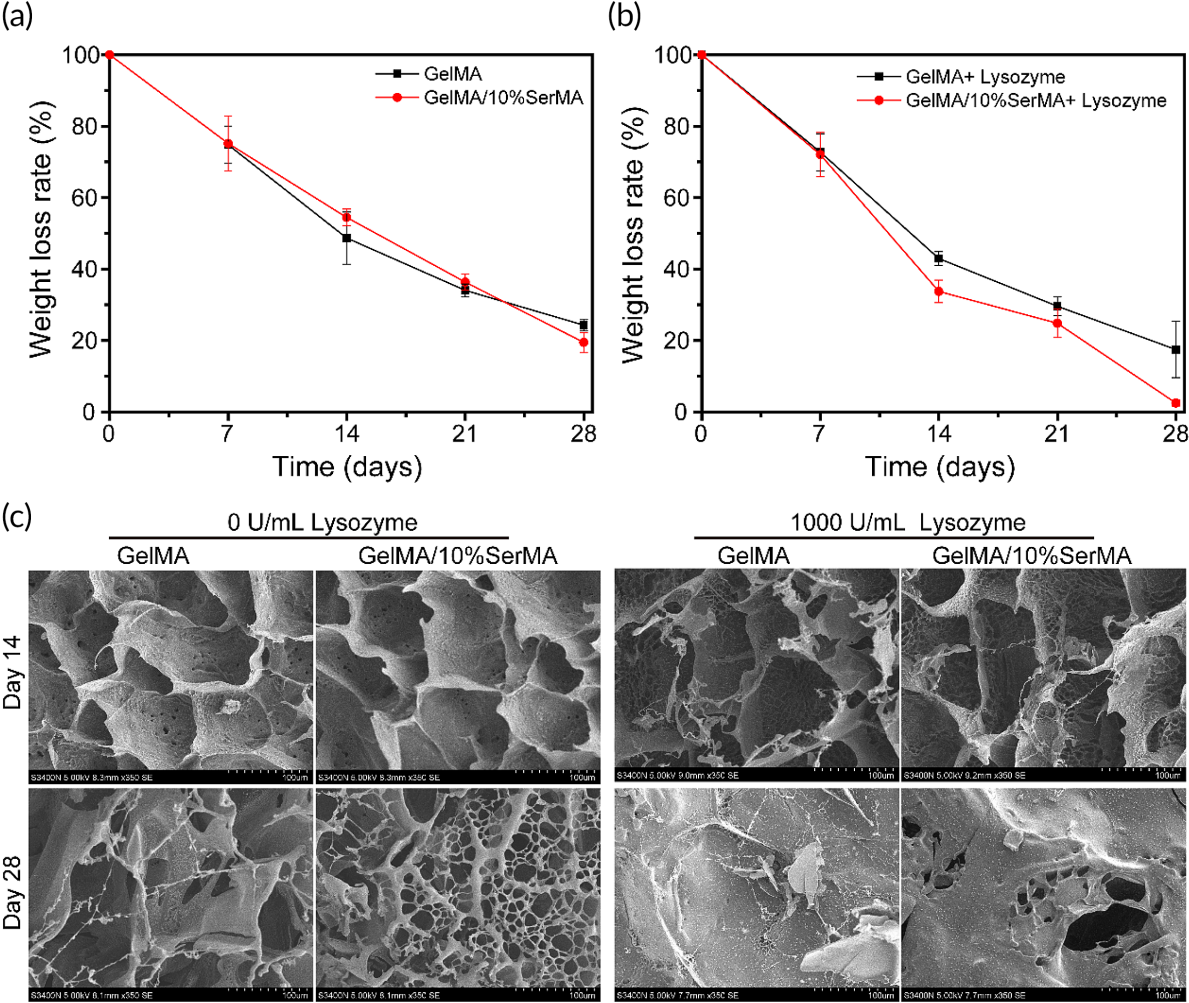
(a) The weight loss ratio of methacrylate gelatin (GelMA) and GelMA/10%SerMA hydrogels in PBS solution (0.01 mol/L, pH = 7.4) with or without 1000 U/ml of lysozyme. (b) SEM images of the surface morphology of GelMA and GelMA/10%SerMA hydrogel at Days 14 and 28

Degradation of biomaterial is one of the most important properties regarding their application in biology, which are directly related to their service life.^48^ The weights of GelMA and GelMA/10%SerMA hydrogels were gradually decreased with the incubation time (Figure 3a,b). Compared to the samples of GelMA hydrogel, the samples of GelMA/10%SerMA hydrogel showed a low weight loss rate, which might due to the higher cross‐linking strength. The same degradation trend also appeared in lysozyme conditions. The hydrogels were rapidly degraded when the samples were subjected to lysozyme solutions. This might be because the gelatin and sericin chains were decomposes by lysozyme.^49^ These results indicated that GelMA/10%SerMA hydrogel exhibited a stable degradation rate and gradually degraded within 4 weeks, which will provide a favorable property for its application in vivo. Meanwhile, the micromorphology of freeze‐dried GelMA and GelMA/10%SerMA hydrogels after degradation was shown in Figure 3c. In the presence of 1000 U/ml lysozyme, cracks and fragments porous structure of hydrogel were increased at Days 14 and 28, which indicated that the hydrogel was degraded with incubation time.

### 3.5 Biocompatible of hydrogel

**FIGURE 4:**
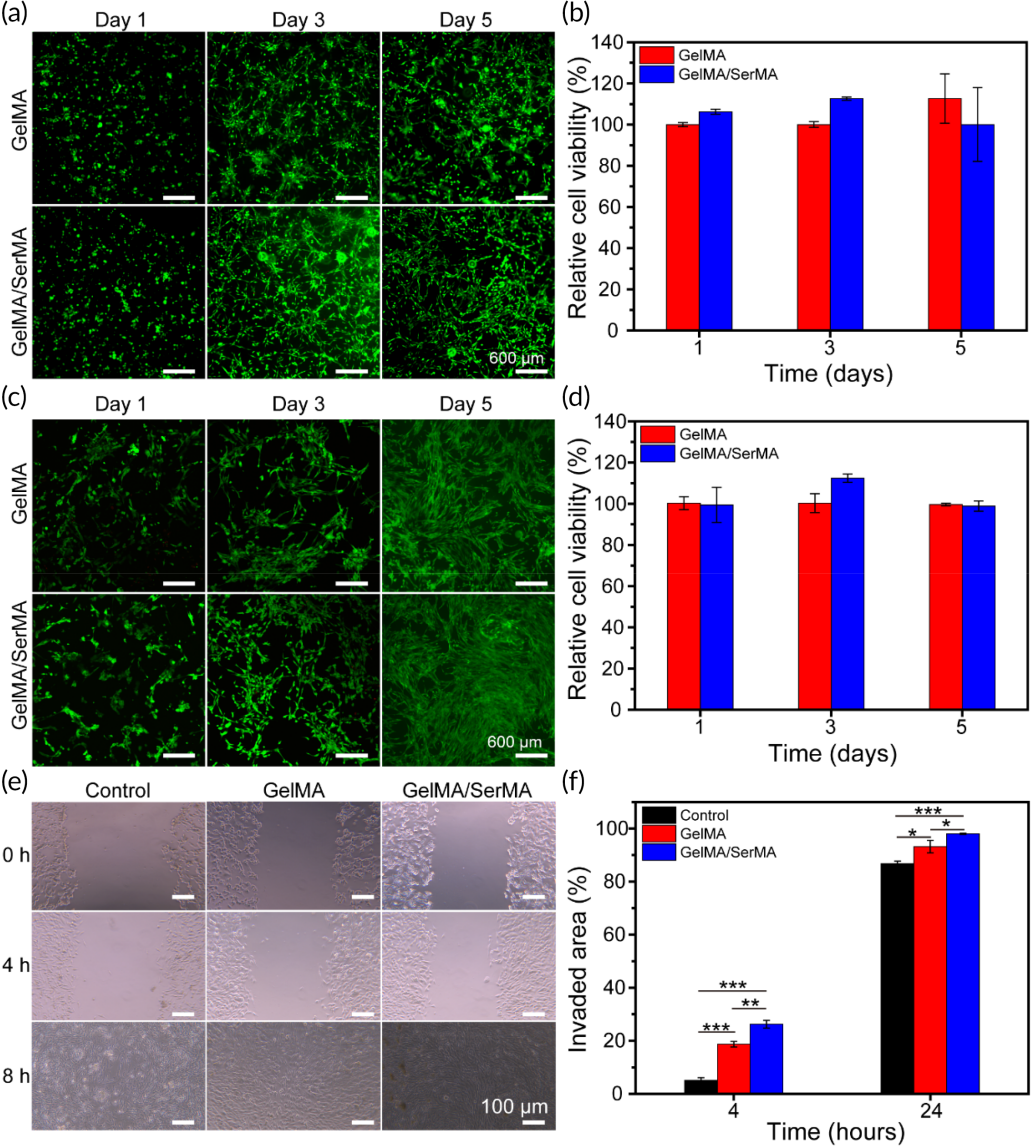
(a) Live/dead staining images of L929 cells cultured on methacrylate gelatin (GelMA), and GelMA/SerMA hydrogels at Days 1, 3, and 5. (b) Cell viability of L929 cells through CCK‐8 assay at Days 1, 3, and 5. (c) Live/dead staining images of human umbilical cord mesenchymal stem cells (HUMSC) cells cultured on GelMA and GelMA/SerMA hydrogels at Days 1, 3, and 5. (d) Cell viability of HUMSC cells through CCK‐8 assay at Days 1, 3, and 5. (e) Photographs showing cell migration in the hydrogel groups at 0, 4, and 8 h. (f) Quantitative analysis of invaded area. * (*p* < 0.05), ** (*p* < 0.01), and *** (*p* < 0.001)

In order to study the biocompatibility of composite hydrogel, we evaluated the survival ability and proliferation effect of L929 and HUMSC cells on the surface of hydrogels for 1, 3, and 5 days. Calcein‐AM/PI staining (green fluorescence represents living cells and red fluorescence represents dead cells) was performed. As shown in Figure 4a,c, it can be seen that L929 and HUMSC cells showed good growth and adhesion on GelMA and GelMA/SerMA hydrogels. After 3 and 5 days of culture, the survival rate of L929 and HUMSC cells in each group was higher than 90% (Figure 4b,d), and there was no significant difference (*p* > 0.05). As shown in Figure S2, the number of HUMSC cells on the GelMA/SerMA hydrogel increased with incubation time. Meanwhile, elongated cells were seen in GelMA/SerMA hydrogel. These data showed that the prepared hydrogels have good biocompatibility and cells can adhere and grow better on GelMA/SerMA hydrogels, which could be used as a biomaterial carrying cells.

The effect of GelMA and GelMA/SerMA on HUMSC cell migration was evaluated (Figure 4e). The culture medium supplemented with the same volume of PBS was used as control. For HUMSC cells, GelMA/SerMA group showed a significant increase at 4 h when compared to the control group. After incubation for 8 h, cells exposed to GelMA/SerMA group showed the highest migration (98.0% ± 0.2%), followed by those exposed to GelMA group (93.2% ± 2.3%) and control group (86.8% ± 1.0%). The increased cell migration rates for cells exposed to GelMA/SerMA hydrogel are due to that sericin proteins could stimulate cell migration through inducing the expression of growth factors including platelet‐derived growth factor (PDGF), vascular endothelial growth factor (VEGF), and insulin‐like growth factor‐1 (IGF‐1).^32^

### 3.6 Cell encapsulation in hydrogel

**FIGURE 5:**
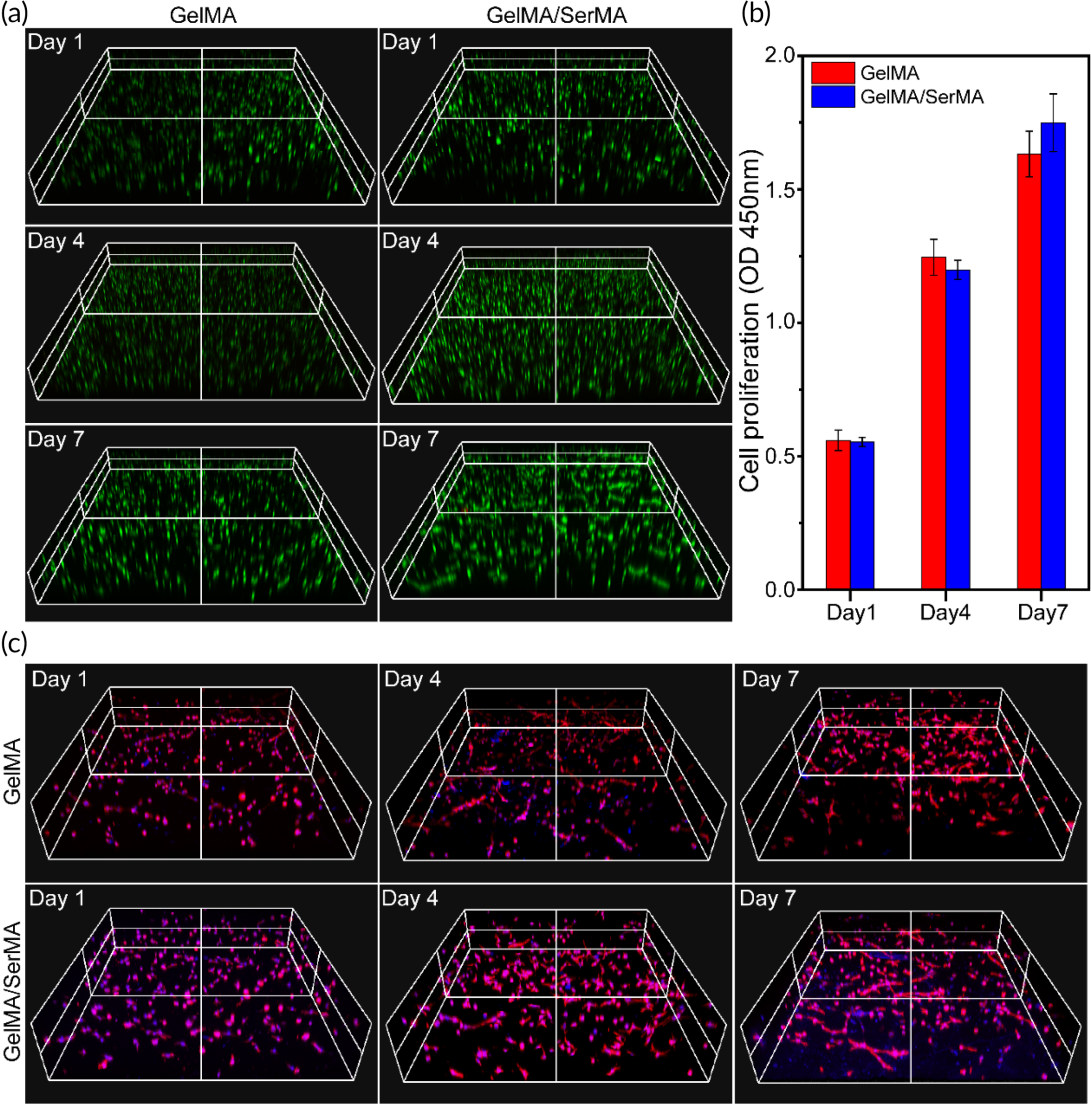
(a) Representative 3D images of encapsulated human umbilical cord mesenchymal stem cells (HUMSC) cells inside methacrylate gelatin (GelMA) and GelMA/SerMA hydrogels. (b) Proliferation of encapsulated HUMSC cells inside GelMA and GelMA/SerMA hydrogels by CCK‐8 assay. (c) Cell morphology of HUMSC cells in GelMA and GelMA/SerMA hydrogels

To further evaluate whether the GelMA and GelMA/SerMA hydrogels can be served effectively as cell carriers, the encapsulation, and culture of HUMSC within hydrogels were performed. After 1, 4, and 7 days of incubation, the overall distribution of cells in hydrogels was uniform and most of the cells were alive (Figure 5a), suggesting that HUMSC cells could survive in the 3D encapsulation process. Next, the proliferation of HUMSC cells in the hydrogel was measured by CCK‐8 method. Figure 5b showed that after the GelMA/SerMA hydrogel encapsulated with HUMSC cells was cultured for 1 day, the optical density (OD) value was 0.55, OD increased to 1.19 for 4 days, and the OD value continued to rise to 1.75 for 7 days. The higher OD value was due to that the gelatin and sericin materials in hydrogel are important ECM, have excellent biocompatibility and contain specific sites that bind to cells, which can promote the adhesion and proliferation of cells. As shown in Figure 5c, the morphology of HUMSC grown on GelMA/SerMA hydrogel was different from that on GelMA hydrogel, which exhibited more elongation and thicker actin filaments. These results suggested that SerMA, as natural polymers, provided a favorable microenvironment for cell adhesion and proliferation, demonstrating that this functionalized sericin is a good candidate material for tissue engineering.

### 3.7 In vivo analyses of endometrial thickness

Next, we established a mice model of endometrial injury through directly injecting 95% ethanol into the uterine horn. After modeling, the mental state of the mice in the model group and the sham operation group was normal, the diet was good and the estrous cycle was regular. As shown in Figure S3, the estrous cycle consists of four stages including proestrus (a number of nucleated epithelial cells, and a small number of keratinized epithelial cells and white blood cells), estrus (a large number of scattered or clustered keratinized enucleated epithelial cells), metestrus (nucleated epithelial cells, enucleated epithelial cells and white blood cells), and diestrus (the cells are mainly white blood cells.).

**FIGURE 6:**
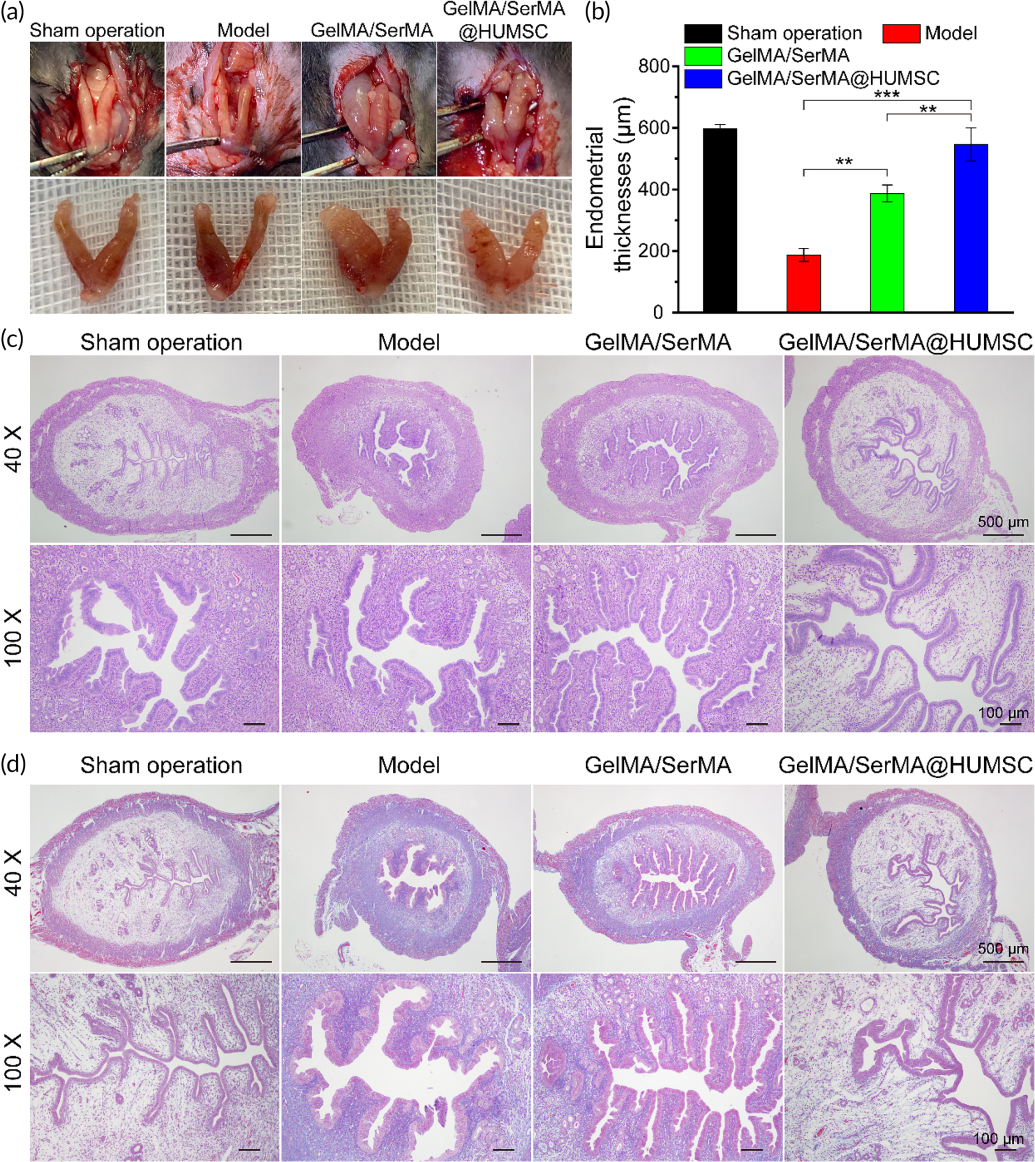
(a) The uterine status and uterine morphology of the different treatment groups (sham operation group, model group, GelMA/SerMA hydrogel group and GelMA/SerMA@HUMSC hydrogel group). (b) Quantitative analysis of endometrial thickness. (c) Representative H&E staining images of uterine samples acquired on the estrous cycles of the mice. (d) Representative Masson's trichrome staining images of uterine samples acquired on the estrous cycles of the mice. * (*p* < 0.05), ** (*p* < 0.01), and *** (*p* < 0.001)

As shown in Figure 6a, the uterine shape of the sham operation group and GelMA/SerMA@HUMSC hydrogel group is regular and uniform and elastic. The uterine shrinkage of the model group is inelastic, dark color, irregular Y shape, and viable necrotic tissue. The damaged uterine tissue showed obvious thinning of the endometrium, which is indistinguishable from the myometrium in some areas. To study the effect of hydrogel on injured endometrium in mice, the uterine specimens of mice in each group were collected and analyzed by HE and Masson staining after three estrous cycles. The suitable thickness of endometrium can provide an excellent attachment place for embryo implantation, which is the key condition to ensure a good pregnancy outcome. The thin endometrium may affect the receptivity of endometrium and lead to the failure of embryo implantation. Therefore, the thickness of endometrium is an important index to measure whether the endometrium is repaired after injury. As shown in Figure 6b, the thickness of endometrium in the sham operation group, Model group, GelMA/SerMA hydrogel group, and GelMA/SerMA@HUMSC hydrogel group were 598.4 ± 12.8, 187.6 ± 20.7, 387.8 ± 27.4, and 546.6 ± 54.2 μm, respectively. Meanwhile, the thickness of endometrium and Gland‐like structures in the GelMA/SerMA@HUMSC hydrogel were similar to that of sham operation group. Notably, the thickness of endometrium in the model group decreased significantly compared with the sham operation group. As shown in Figure 6c, the uterine cavity blockage and adhesion in the GelMA/SerMA@HUMSC hydrogel treatment group were significantly improved compared with the model group, and the thickness of the endometrium was significantly thicker than that in the model group (*p* < 0.001). As shown in Figure 6d, the uterine tissue of the model group has numerous fibrillates. However, the fibrotic area in the GelMA/SerMA@HUMSC hydrogel treatment group showed small amounts of fibrillates, which was attributed to that HUMSC could inhibit endometrial fibrosis.^50^ Transforming growth factor (TGF‐β1) was a multifunctional polypeptide cytokine, which could promote fibroblast proliferation and fibrin formation at the high expression levels, resulting in the development of endometriosis. As shown in Figure S4A,B, the expression of TGF‐β1 in the GelMA/SerMA@HUMSC hydrogel treatment group was the lowest, which indicated GelMA/SerMA@HUMSC hydrogel could improve fibrosis of endometriosis. These results showed that GelMA/SerMA@HUMSC hydrogel transplantation group could increase the thickness of endometrium and improve the endometrial interstitial fibrosis, resulting in promoting the repair of injured endometrium.

### 3.8 Immunofluorescence staining

Uterine receptivity is regulated by a number of factors that coordinate intrauterine changes facilitate blastocyst implantation. Next, we evaluated the expression of differentiation 31 (CD31), cytokeratin 18 (CK18), cell proliferation antigen Ki‐67 (Ki67), Vimentin, and Caspase 3, which are widely studied markers of endometrial receptivity and play multiple roles in a series of related developmental processes.

**FIGURE 7:**
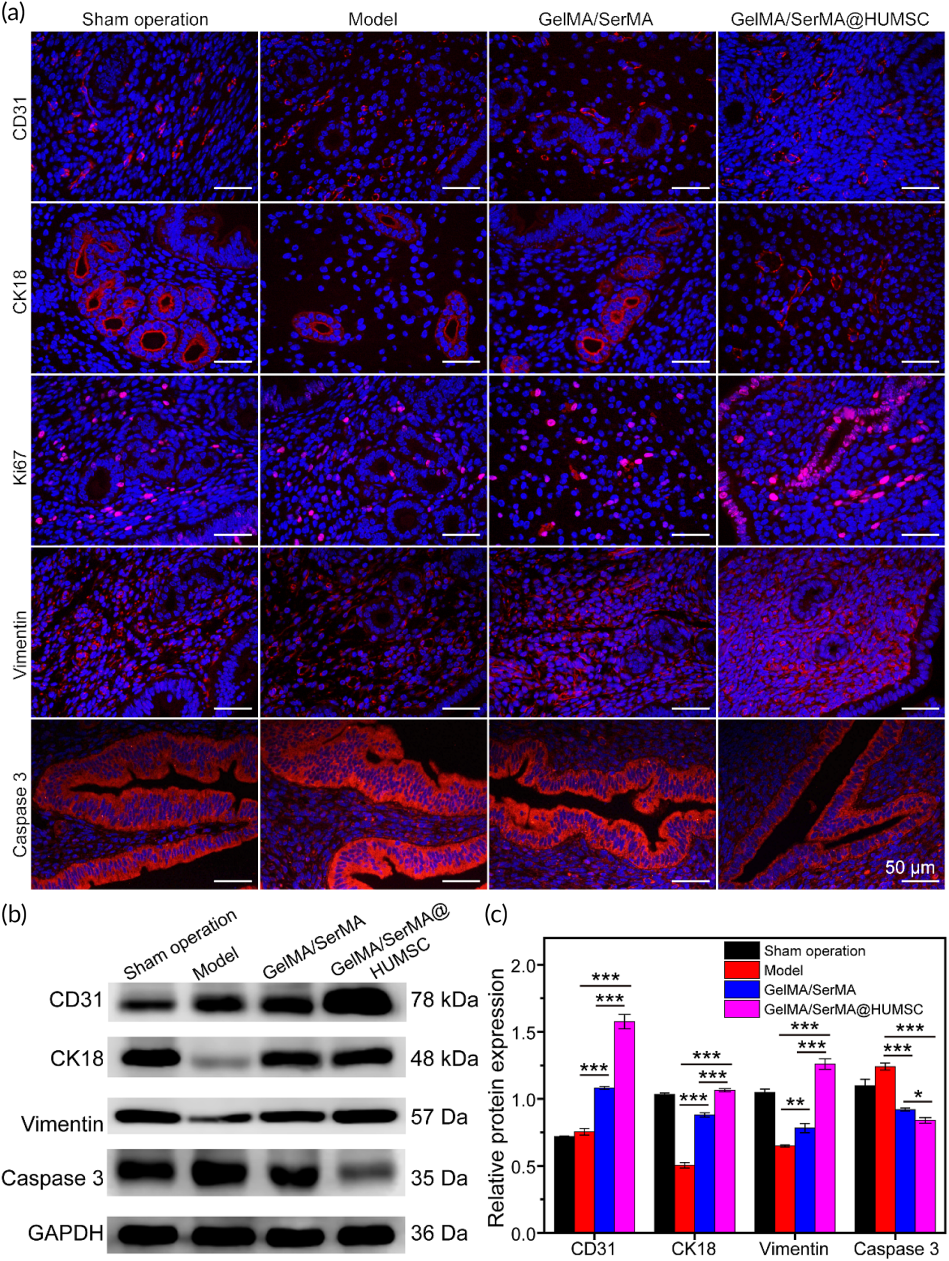
(a) Representative CD31, CK18, Ki67, Vimentin, and Caspase 3 images of immunofluorescence staining of uterine samples. (b) Western bolt analysis of CD31, CK18, Vimentin, and Caspase 3. (c) The quantitative analysis of WB data. * (*p* < 0.05), ** (*p* < 0.01), and *** (*p* < 0.001)

CD31 is an important index to evaluate neovascularization.^51^ As shown in Figure 7a, the distribution of CD31 in the sham operation group was more than that in the model group, while the expression of CD31 in the GelMA/SerMA hydrogel treatment group was between the sham operation group and the model group. As shown in Figure S5A, the CD31 positive ratio of sham operation group, model group, GelMA/SerMA hydrogel group, and GelMA/SerMA@HUMSC hydrogel group were 8.2% ± 1.0%, 15.4% ± 1.4%, 13.8% ± 0.6%, and 23.9% ± 2.5%, respectively. CK18 is member of the keratin family of intermediate filament proteins, which is more expressed in the cytoplasm of endometrial epithelial cells.^52^ As shown in Figure S5B, the expression of CK18 in the sham operation group is more than that in the model group, while that in the model group is less. The expression of CK18 in the GelMA/SerMA hydrogel treatment group and the GelMA/SerMA@HUMSC hydrogel treatment group is more than that in the model group, indicating that the efficacy in the hydrogel treated group is better than that in the model group.

Ki67 is an important index to evaluate cell proliferation.^53^ There is more proliferation in the sham operation group and less in the model group. As shown in Figure S5C, the expression of Ki67 in the GelMA/SerMA hydrogel treatment group and the GelMA/SerMA@HUMSC hydrogel treatment group is more than that in the model group, indicating better cell proliferation and tissue growth. Vimentin is an indicator of the specific expression of Vimentin in endometrial stromal cells.^54^ As shown in Figure S5D, the expression of CK18 in the sham operation group is more than that in the model group, while the expression of CK18 in the GelMA/SerMA hydrogel treatment group and the GelMA/SerMA@HUMSC hydrogel treatment group is more, and the expression effect is the same as that in the sham operation group, indicating that the combination of hydrogels with HUMSC may achieve better therapeutic effect. Caspase‐3 activation is a hallmark of apoptotic cell death. As shown in Figure S5E, the expression of Caspase 3 in GelMA/SerMA hydrogel treatment group and the GelMA/SerMA@HUMSC hydrogel treatment group. As shown in Figure 7b,c, western blot analysis showed a similar trend.

### 3.9 Fertility evaluation

**FIGURE 8:**
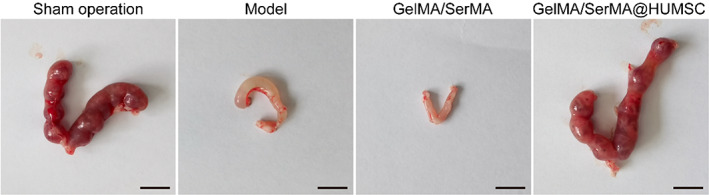
Pregnancies in the different treatment groups (sham operation group, model group, GelMA/SerMA hydrogel group and GelMA/SerMA@HUMSC hydrogel group)

To study the effect of hydrogel transplantation on the fertility of mice with endometrial injury, hydrogel was transplanted into the uterine cavity of mice after modeling, and female mice and male mice were mated in cages after three estrous cycles. As shown in Figure 8, the implantation number was the highest in the sham operation group after cage mating. Compared with the model group, the implantation number in the GelMA/SerMA@HUMSC hydrogel group was significantly higher than those in the model group. All data showed GelMA/SerMA@HUMSC hydrogel can promote functional endometrial regeneration and facilitate the recovery of fertility, which could regulate endometrial regeneration through the use of multifunctional hydrogels. Prior study demonstrated that stem cell transplantation can improve the fertility of rats with damaged endometrium and support fertilized egg implantation and embryonic development.^50^

## 4 CONCLUSION

In this study, we have developed an injectable hydrogel based on methacrylate gelatin (GelMA) and methacrylate sericin (SerMA) matrix as cell delivery carriers of HUMSC to promote angiogenesis and endometrial regeneration, thus promoting the recovery of fertility. The swelling, mechanical, and degradation properties of GelMA/SerMA hydrogel could be regulated by adjusting the concentration of SerMA. In vitro results showed that the GelMA/SerMA hydrogel could promote the proliferation and migration of HUMSC and have good cell encapsulation ability. On mice endometrial damage model in vivo, the GelMA/SerMA@HUMSC hydrogel could increase the thickness of endometrium and improve endometrial interstitial fibrosis through upregulating the expression of CD31, CK18, Ki67 and Vimentin, and downregulating the expression of Caspase 3, resulting in promoting the repair of injured endometrium. Therefore, local use of hydrogel with stem cell encapsulation is a promising method to enhance endometrial regeneration and improve pregnancy outcome.

## AUTHOR CONTRIBUTIONS

**Lixuan Chen:** Conceptualization (equal); methodology (equal); writing ‐ original draft (equal). **Ling Li:** Methodology (equal); writing ‐ original draft (equal). **Qinglin Mo:** Data curation (equal); investigation (equal); methodology (equal). **Xiaomin Zhang:** Data curation (equal); formal analysis (equal); methodology (equal). **Chaolin Chen:** Data curation (equal); formal analysis (equal); methodology (equal). **Yingnan Wu:** Formal analysis (equal); methodology (equal); software (equal). Xiaoli Zeng: Methodology (equal); resources (equal). **Kaixian Deng:** Data curation (equal); methodology (equal); resources (equal); **Nanbo Liu:** Data curation (equal); methodology (equal); resources (equal). **Ping Zhu, Mingxing Liu and Yang Xiao:** Conceptualization, Methodology, Formal analysis, Data curation, Writing ‐ review & editing, Supervision, Funding acquisition.

## CONFLICT OF INTERESTS

The authors declare no competing financial interest.

### PEER REVIEW

The peer review history for this article is available at https://publons.com/publon/10.1002/btm2.10328.

## Supporting information


**Appendix S1** Supporting Information

## Data Availability

Some or all data, models, or code generated or used during the study are available from the corresponding author by request.
