# Supplementary material for: An injectable gelatin/sericin hydrogel loaded with human umbilical cord mesenchymal stem cells for the treatment of uterine injury
Source: Bioeng Transl Med. 2022 May 18;8(1):e10328. doi: 10.1002/btm2.10328 (PMC9842051; doi:10.1002/btm2.10328)
Supplement: Supplementary file 1 — Appendix S1 Supporting Information [file BTM2-8-e10328-s001.docx]

**Supporting Information**

**An** **injectable gelatin / sericin hydrogel loaded with human umbilical cord mesenchymal stem cells for the treatment of uterine injury**

Lixuan Chen^a,b,1^, Ling Li^c,1^, Qinglin Mo^d^, Xiaoming Zhang^a,b^, Chaolin Chen^d^, Yingnan Wu^d^, Xiaoli Zeng^e^, Kaixian Deng^f^, Nanbo Liu^g^, Ping Zhu^g,*^, Mingxing Liu^h,*^, Yang Xiao^a,i,*^

1. Guangzhou University of Chinese Medicine, Guangzhou, Guangdong, 510006, China.
2. Jinshazhou Hospital of Guangzhou University of Chinese Medicine, Guangzhou, Guangdong, 510168, China.
3. Jiangmen Maternity and Child Health Care Hospital, Jiangmen, Guangdong, 529000, China.
4. Translational Medicine Center, The Second Affiliated Hospital of Guangzhou Medical University, Guangzhou, Guangdong, 510260, China.
5. National Seed Cell Bank of South China for Tissue Engineering, Guangzhou, Guangdong, 510663, China.
6. Department of Gynecology, Shunde Hospital, Southern Medical University (The First People's Hospital of Shunde), Foshan, Guangdong, 528308, China.

g. Guangdong Cardiovascular Institute, Guangdong Provincial People's Hospital, Guangdong Academy of Medical Sciences, Guangzhou 510100, China.

h. Department of Obstetrics and Gynecology, Key Laboratory for Major Obstetric Diseases of Guangdong Province, The Third Affiliated Hospital of Guangzhou Medical University, Guangzhou, Guangdong, 510150, China.

i. Shenzhen Qianhai Shekou Pilot Free Trade Zone Hospital No.36, 7th Industrial Road, Shekou, Shenzhen, Guangdong, 518067, China.

^1^ These authors contributed equally to this work.

^*^Corresponding authors. *E-mail addresses*: [tanganqier@163.com](mailto:tanganqier@163.com) (Ping Zhu); 2009683032@gzhmu.edu.cn (Mingxing Liu); jdxiao111@163.com (Yang Xiao).

**

**

**Figure S1.** The pore size of different hydrogel groups.


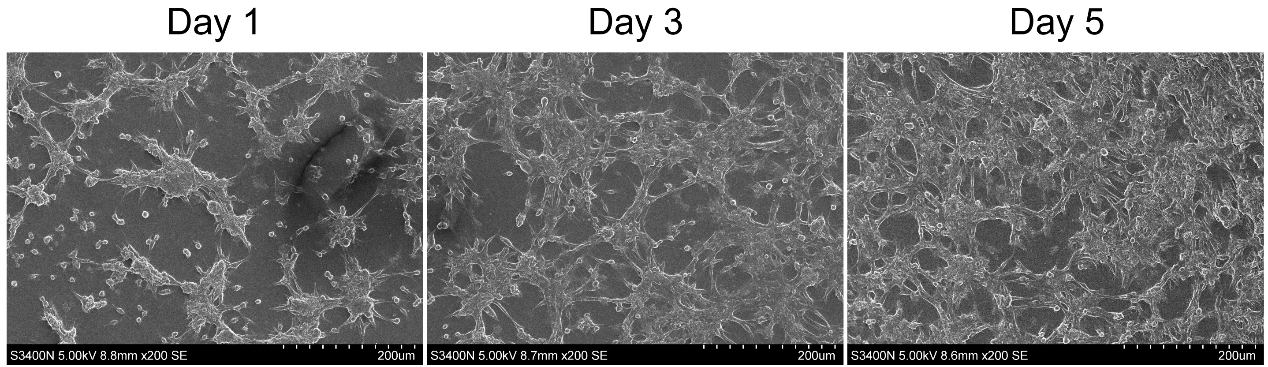


**Figure S2.** SEM of HUMSC cultured on GelMA/SerMA hydrogels versus different culture times

**
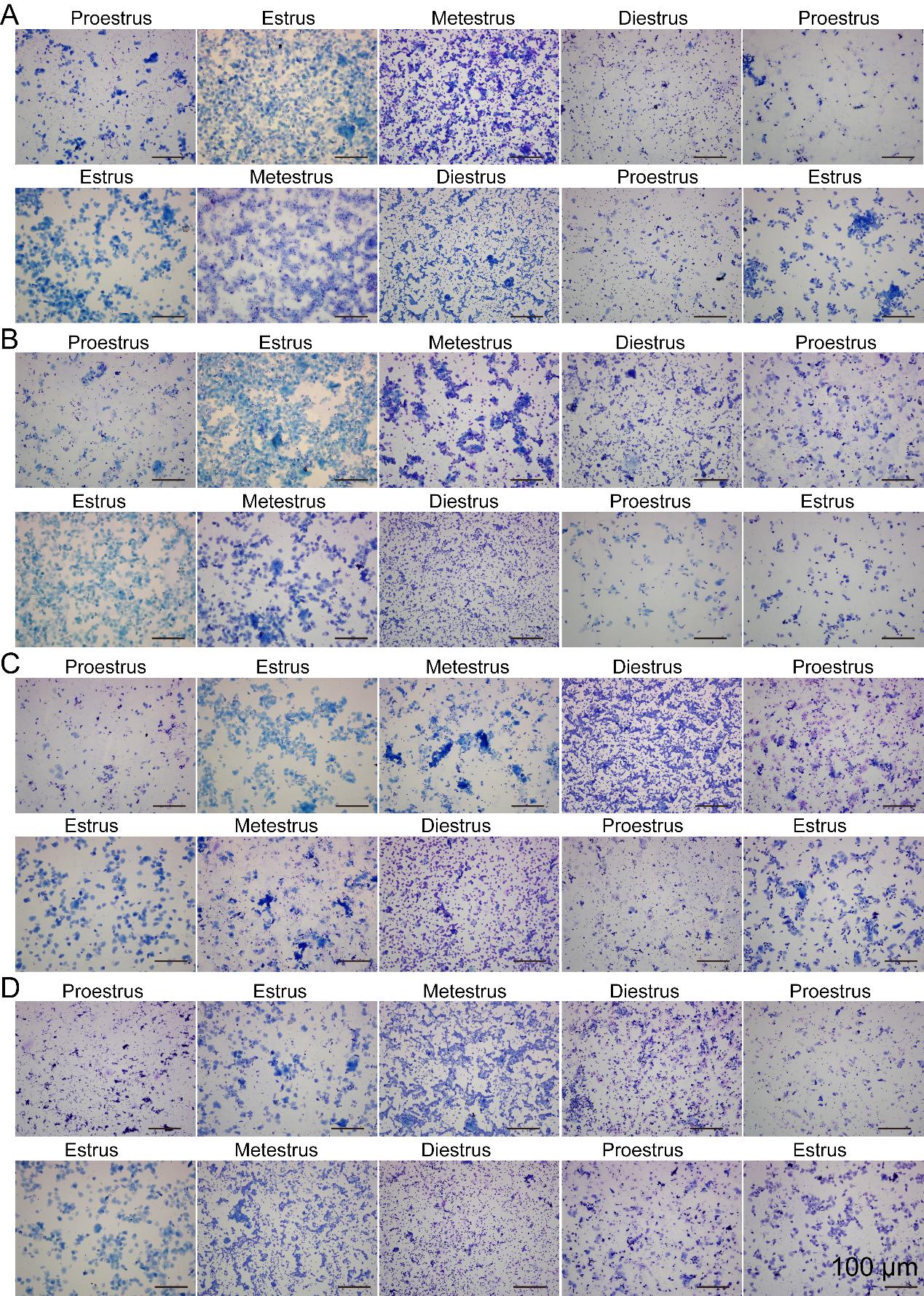
**

**Figure S3.** The estrous cycles of (A) sham operation group, (B) model group, (C) GelMA/SerMA hydrogel group and (D) GelMA/SerMA@HUMSC hydrogel group. **
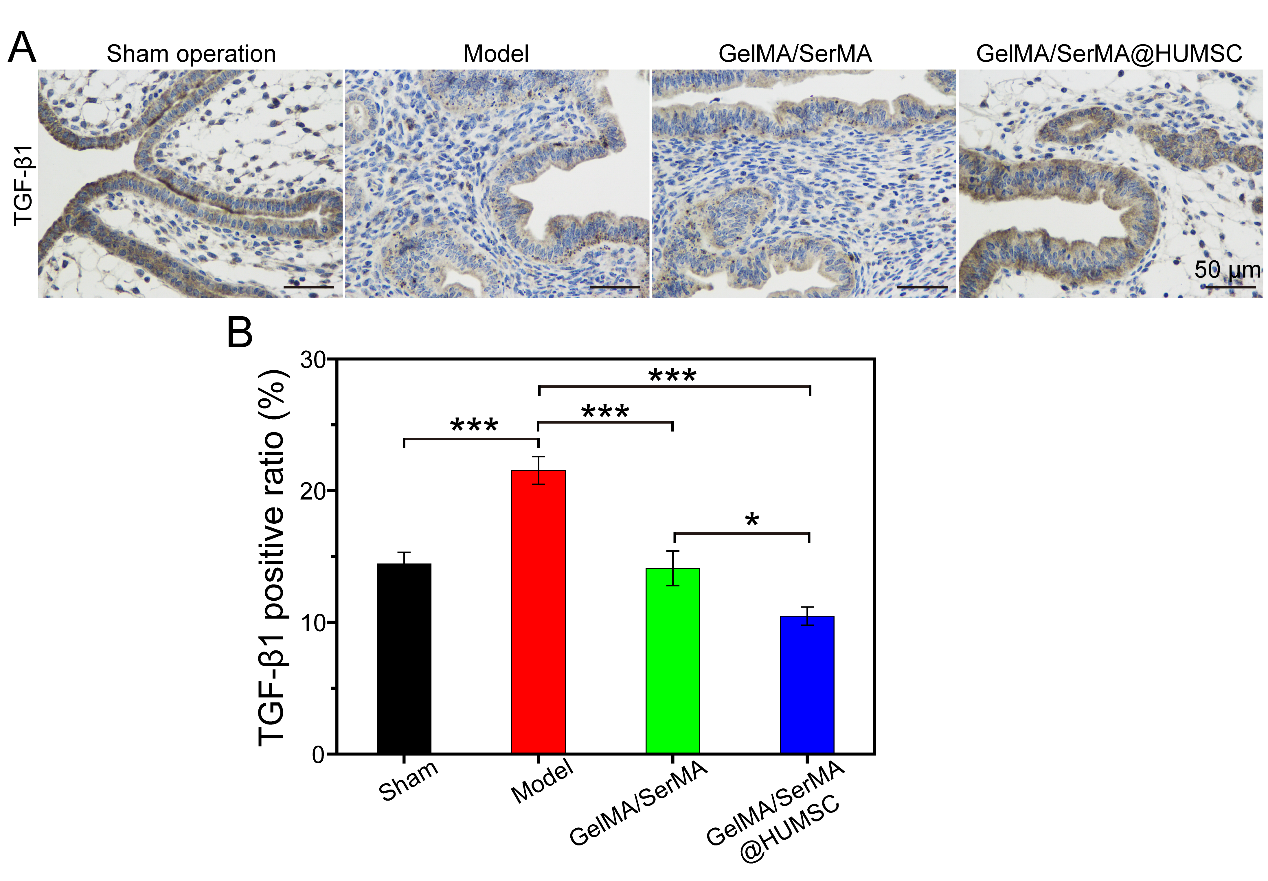
**

**Figure S4.** (A) Representative TGF-β1 images of immunohistochemical staining of uterine samples. (B) Quantitative analysis of TGF-β1 positive ratio. * (p＜0.05), ** (p＜0.01), and *** (p＜0.001).


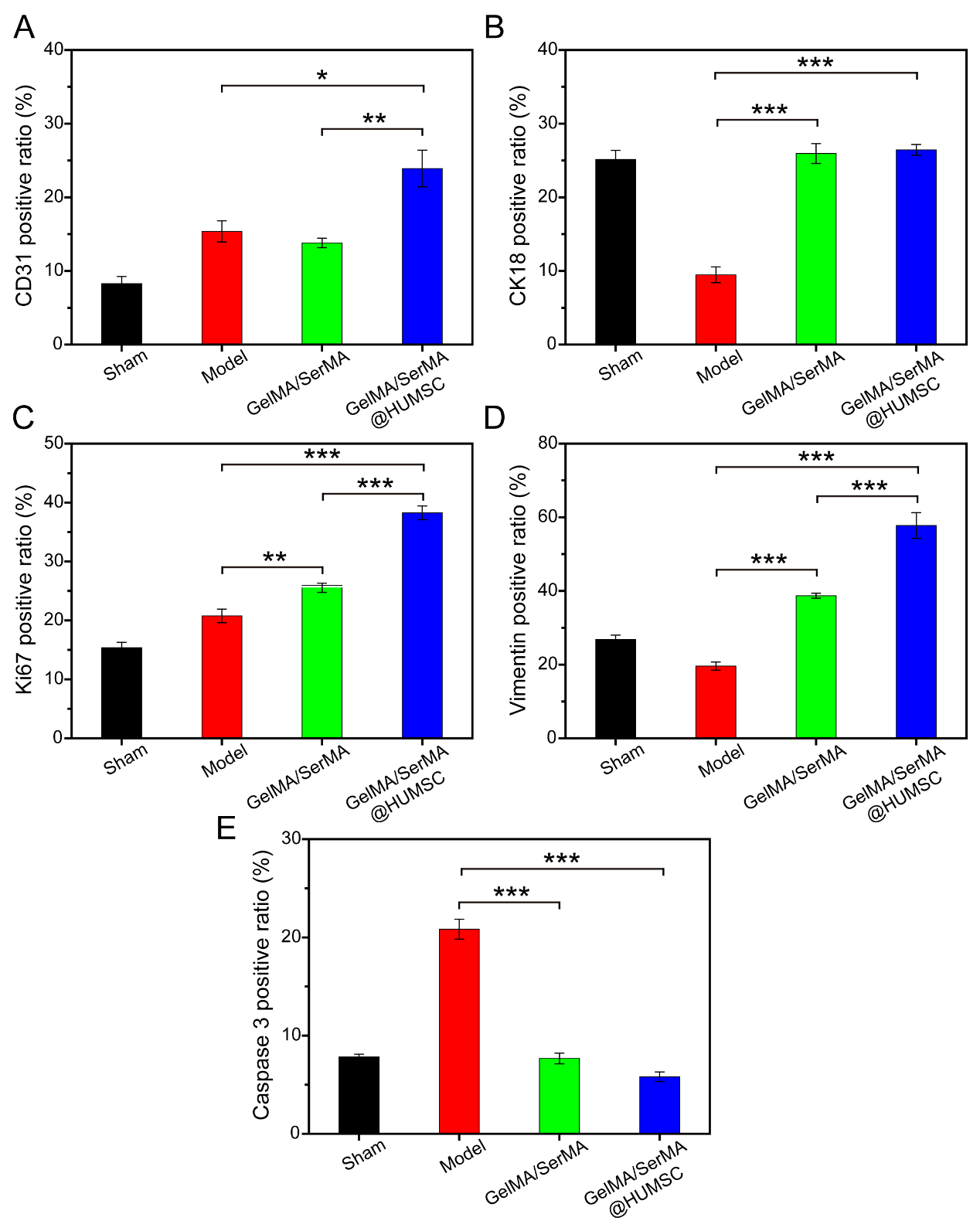


**Figure S5.** Quantitative analysis of CD31 positive ratio (A), CK18 positive ratio (B), Ki67 positive ratio (C), Vimentin positive ratio (D) and Caspase 3 positive ratio (E).
